# α-Linolenic Acid-Rich Diet Influences Microbiota Composition and Villus Morphology of the Mouse Small Intestine

**DOI:** 10.3390/nu12030732

**Published:** 2020-03-11

**Authors:** Hristo Todorov, Bettina Kollar, Franziska Bayer, Inês Brandão, Amrit Mann, Julia Mohr, Giulia Pontarollo, Henning Formes, Roland Stauber, Jens M. Kittner, Kristina Endres, Bernhard Watzer, Wolfgang Andreas Nockher, Felix Sommer, Susanne Gerber, Christoph Reinhardt

**Affiliations:** 1 Institute for Developmental Biology and Neurobiology, Faculty of Biology and Center for Computational Sciences in Mainz, Johannes Gutenberg-University Mainz, Staudingerweg 9, 55128 Mainz, Germany; hristo_td@yahoo.com (H.T.); gerber.sj@gmail.com (S.G.); 2 Fresenius Kabi Deutschland GmbH, Borkenberg 14, 61440 Oberursel, Germany; 3 Center for Thrombosis and Hemostasis (CTH), University Medical Center Mainz, Johannes Gutenberg-University Mainz, Langenbeckstrasse 1, 55131 Mainz, Germany; kollar.lb@gmail.com (B.K.); inesm.brandao@gmail.com (I.B.); amrit.mann@unimedizin-mainz.de (A.M.); jumohr@students.uni-mainz.de (J.M.); giulia.pontarollo@unimedizin-mainz.de (G.P.);; 4 Centro de Apoio Tecnológico Agro Alimentar (CATAA), Zona Industrial de Castelo Branco, Rua A, 6000-459 Castelo Branco, Portugal; 5 Nanobiomedicine, University Medical Center Mainz, Johannes Gutenberg-University Mainz, Langenbeckstrasse 1, 55131 Mainz, Germany; rstauber@uni-mainz.de; 6 Medical Department 2 (Gastroenterology, Hepatology, Pneumology, Endocrinology) Klinikum Darmstadt GmbH, Grafenstr. 9, 64283 Darmstadt, Germany; jens.kittner@mail.klinikum-darmstadt.de; 7 Department of Psychiatry and Psychotherapy, University Medical Center of the Johannes Gutenberg-University Mainz, 55131 Mainz, Germany; 8 Metabolomics Core Facility, Philipps-University, 35043 Marburg, Germany; watzer@staff.uni-marburg.de; 9 Institute of Laboratory Medicine and Pathobiochemistry, Philipps-University, 35043 Marburg, Germany; andreas.nockher@uk-gm.de; 10 Institute of Clinical Molecular Biology, Christian-Albrechts-University Kiel, 24105 Kiel, Germany; f.sommer@ikmb.uni-kiel.de; 11 German Center for Cardiovascular Research (DZHK), Partner Site RheinMain, 55131 Mainz, Germany

**Keywords:** α-linolenic acid, microbiota, epithelial renewal, goblet cells, paneth cells, villus morphology

## Abstract

α-Linolenic acid (ALA) is well-known for its anti-inflammatory activity. In contrast, the influence of an ALA-rich diet on intestinal microbiota composition and its impact on small intestine morphology are not fully understood. In the current study, we kept adult C57BL/6J mice for 4 weeks on an ALA-rich or control diet. Characterization of the microbial composition of the small intestine revealed that the ALA diet was associated with an enrichment in *Prevotella* and *Parabacteroides*. In contrast, taxa belonging to the Firmicutes phylum, including *Lactobacillus*, *Clostridium* cluster XIVa, Lachnospiraceae and *Streptococcus*, had significantly lower abundance compared to control diet. Metagenome prediction indicated an enrichment in functional pathways such as bacterial secretion system in the ALA group, whereas the two-component system and ALA metabolism pathways were downregulated. We also observed increased levels of ALA and its metabolites eicosapentanoic and docosahexanoic acid, but reduced levels of arachidonic acid in the intestinal tissue of ALA-fed mice. Furthermore, intestinal morphology in the ALA group was characterized by elongated villus structures with increased counts of epithelial cells and reduced epithelial proliferation rate. Interestingly, the ALA diet reduced relative goblet and Paneth cell counts. Of note, high-fat Western-type diet feeding resulted in a comparable adaptation of the small intestine. Collectively, our study demonstrates the impact of ALA on the gut microbiome and reveals the nutritional regulation of gut morphology.

## 1. Introduction

The ω-3 polyunsaturated fatty acid (PUFA) α-linolenic acid (ALA, 18:3 n-3) is an essential plant-derived fatty acid that is abundant in oil produced from perilla, linseed, rapeseed and soy. This macronutrient exerts anti-inflammatory properties through the generation of oxylipins [1]. ALA interferes with the arachidonic acid (AA) metabolism and inhibits the prostaglandin biosynthesis pathway, thereby reducing the concentration of pro-inflammatory oxylipins [2]. In addition to its effects on the formation of anti-inflammatory mediators [3,4] and its anti-hypertensive action [5,6,7], there is increasing evidence that ALA plays a role in ameliorating intestinal inflammatory disease phenotypes [8,9,10,11]. Furthermore, ALA-rich diets were reported to protect from the development of colon carcinomas [12].

In contrast to the recognized role of ALA in inflammatory bowel disease, information on the influence of this essential PUFA in normal gut homeostasis and its interplay with the commensal gut microbiota remains sparse. Nutritional studies on rats fed with perilla oil, a source rich in ALA, indicated a decrease in the Firmicutes to Bacteroidetes ratio and an increase in the abundance of Spirochaetes in the perilla oil group relative to normal lab chow [13]. A study in mice showed that flaxseed/fish oil feeding rich in ω-3 PUFA promoted the growth of *Bifidobacterium* and improved metabolic outcome, indicated by reduced liver weight and hepatic triglyceride concentration compared to palm oil diet [14]. One of the possible mechanisms by which PUFA might beneficially impact host metabolism is through the production of conjugated fatty acids by intestinal bacteria. Research so far has mainly focused on conjugated linoleic acid which is an intermediate metabolite in the saturation pathway of the ω-6 PUFA linoleic acid [15]. However, conjugated isomers of ALA have also gained attention due to their reported anti-inflammatory, anti-carcinogenic and anti-obesogenic properties [16,17,18]. In vitro studies have shown that certain strains of Bifidobacteria [19,20], Propionibacteria [20] and lactic acid bacteria [21,22] are able to metabolize ALA to conjugated ALA isomers. Furthermore, Druart et al. demonstrated that the commensal gut microbiota contributes to the production of PUFA-derived metabolites in vivo by reporting increased colonic contents of conjugated linoleic acid isomers and non-conjugated metabolites in conventionalized compared to germ-free mice [23]. Ohue-Kitano and colleagues reported that short-term feeding of C57BL/6 mice with ALA and the ALA-derived metabolites of intestinal lactic acid bacteria affect intestinal immune homeostasis [24]. The authors showed that ALA and its metabolite 13-hydroxy-9(*Z*),15(*Z*)-octadecadienoic acid promote the accumulation of anti-inflammatory M2 macrophages in the small intestinal lamina propria. Additionally, PUFA-rich diets may impact the differentiation of the intestinal epithelial lineage as gastric gavage in rat pups with rapeseed oil and sunflower oil, which are both rich in PUFA, decreased mucus secreting goblet cell numbers in the colon [25]. Intestinal epithelial cells originate from stem cells located at the base of the Lieberkühn crypt and differentiate along the crypt-villus axis. To date, it is not completely clear how ALA shifts intestinal microbiota composition. Additionally, investigations on the influence of PUFAs on small intestinal morphology and renewal of the gut epithelial lineage under steady-state conditions are sparse [26].

In the current study, we hypothesized that an ALA-rich diet leads to compositional changes in the commensal microbiota of the mouse small intestine. Furthermore, we investigated the potential impact of increased dietary amounts of ALA on gut morphology in the mid small intestine.

## 2. Materials and Methods

### 2.1. Animals

Male C57BL/6J mice that were 10–14 weeks old were obtained from the Jackson Laboratory. Animals were held at the Translational Animal Research Center (TARC) of the University Medical Center Mainz under specific pathogen-free (SPF) conditions in EU Type II individually ventilated cages under constant room temperature and air humidity with a 12 h light-dark cycle. Mice had ad libitum access to water and autoclaved chow. The animals were assigned to a control standard diet group (Altromin Spezialfutter GmbH & Co. KG, Lage, Germany) and to an α-linolenic acid rich diet group (standard Altromin diet + 20% perilla oil). The composition and fatty acid profile of both diets is shown in Table 1. To test the specificity of ALA dietary effects on the gut morphology, we fed an additional group of 7 mice with a pro-inflammatory, cholesterol-rich high-fat Western-type diet (TD.88137, Envigo, Venray, Netherlands). The composition and fatty acid profile of this diet are shown in Table 2. After a 4 week treatment period, mice were sacrificed via cervical dislocation. The small intestine was collected and cut into eight equivalent pieces. Segment 5 was used for all subsequent experiments. We refer to this segment as the mid small intestine or jejunum.

All animal experiments were approved by the Institutional Animal Care and Use Committee of Rhineland-Palatinate (23177-07/G13-1-072; 23177-07/G16-1-013). The authors confirm that all experiments were performed in accordance with relevant guidelines and regulations. 

### 2.2. Histological Analysis of the Small Intestine

#### 2.2.1. Proliferation Assay

The fifth segment of eight equally sized segments of the small intestine was flushed with cold PBS and fixed in Roti^®^-Histofix (#P087, Roth) at 4 °C overnight. Tissue was processed for paraffin embedding at the Core Facility Histology at University Medical Center Mainz. Tissue sections were cut at 3 µm thickness, dewaxed and heat-induced epitope-retrieval was done using citrate buffer (10 mM sodium citrate, pH 6.6). Unspecific binding was blocked using normal goat serum (#S-1000, Vector Laboratories, 5% *v*/*v* in PBS) and sections were incubated for 1 h at room temperature with rabbit anti mouse-Ki-67 antibody (1:500 in blocking solution, #IHC-00375, Bethyl Laboratories Inc.). After washing, sections were incubated with the biotinylated anti-rabbit antibody (#BA-1000, Vector Laboratories) and signal detection was done using Vectastain^®^ Elite^®^ ABC HRP (#PK-7100, Vector Laboratories) and 3,3’-diaminobenzidine (DAB) as substrate according to the manufacturer’s protocol. Sections were dehydrated and mounted using Eukitt^®^ mounting medium (#SIAM03989, VWR). Ki67-positive cells as well as total number of cells per villus/crypt were counted, averaged and represented as percent.

#### 2.2.2. Periodic Acid-Schiff (PAS) Staining

Paraffin slides were stained for analysis of intestinal tissue morphology using PAS staining. Briefly, the hydrated slides were oxidized with periodic acid for 5 min, washed with distilled water 4–5 times and subsequently incubated with Schiff reagent for 15 min at room temperature. The stained slides were then washed for 5 min under tap water and counterstained in hemalaun. Next, slides were held under flowing water until the counterstain was blue. Finally, slides were dehydrated by incubation in ethanol in increasing concentrations (3 min in 30% ethanol, 50% ethanol, 70% ethanol, 90% ethanol, then 2x 5 min in xylol) followed by mounting in Eukitt mounting medium (#SIAM03989, VWR).

**Figure 1:**
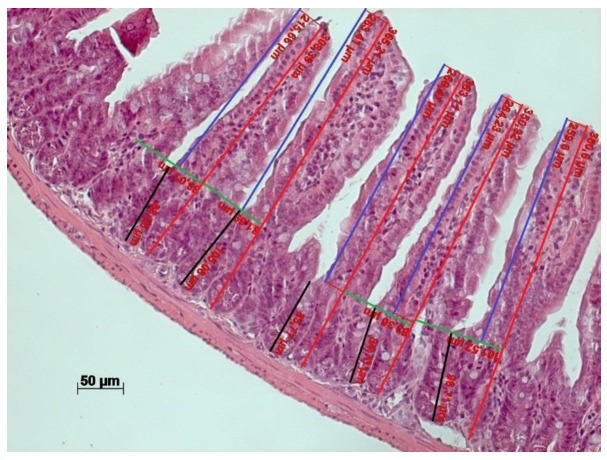
Schematic representation of measurements of intestinal morphology parameters. The vertical red lines correspond to mucosal thickness, blue lines indicate villus length, black lines indicate crypt depth and horizontal green lines demonstrate how villus spacing was determined.

Villus morphology of the small intestine was evaluated by inspecting at least 10 villus structures per cross section from 6–7 animals per group. We determined the average number of epithelial cells per villus, mucosal thickness, crypt depth, villus length and villus spacing for each animal. Villus spacing was calculated by measuring the distance from the center of one villus at the villus base to the center of the next villus. We calculated this measure for at least 10 pairs of villi and took the average value as the final estimate. Furthermore, we determined the percentage of Paneth cells and goblet cells relative to all epithelial cells. Figure 1 shows a schematic representation of how different morphological parameters were measured.

### 2.3. GC-MS/MS Quantification of Fatty Acids in Intestinal Tissue and Diet

Fatty acid (FA) extraction, derivatization and analysis of the intestine samples was performed with slight modifications as previously described [27]. For FA determination, samples of 25–125 mg lyophilized intestinal tissue were used (*n* = 10 in the ALA group and *n* = 9 in the control diet group). The samples were dissolved in 5 mL of a mixture of hexane/isopropanol 3:2 (v/v; VWR International GmbH, Darmstadt, Germany; LC-MS grade) spiked with 1 μg of heptadecenoic acid (cis-10; Larodan, Malmö, Sweden) as internal standard. Then, 3 mL of a 6.7% Na_2_SO_4_ solution (Merck KGaA, Darmstadt, Germany) was added. From the centrifuged mixture, the supernatant hexane phase was removed and evaporated to dryness under a gentle stream of nitrogen. A solution of 14% boron trifluoride (BF3) in methanol (Sigma-Aldrich, Hamburg, Germany) was added and incubated at 100 °C for 10 min for complete esterification. The formed FA-methyl esters were extracted after addition of 1 mL water and 3 mL hexane then evaporated under nitrogen and resolved in 500 μL of hexane. To determine the FAs, a 1 µL aliquot of the hexane solution was injected into a Triple Quad GC-MS/MS (Agilent 7000) system. A multiple reaction monitoring (MRM) method was used for analyte detection. Individual FA concentrations were calculated as relative percentage with an evaluated FA reference standard set (GLC-744, Nu-Chek Prep, Inc. Elysian, MN, USA) at 100% or as absolute values. Mass Hunter Quant 5.0 and Mass Hunter Qual 5.0 software were used for data analysis.

The fatty acid profile of the control and ALA-rich diets was determined using the methods described above. The amount of each fatty acid relative to all fatty acids was determined in technical triplicates for each diet and the average value was reported. The amount of each fatty acid as percentage from the diet (as reported in Table 1) was obtained by multiplying the experimentally measured amount with the total relative amount of fat in the control and ALA-rich diet, respectively.

### 2.4. Microbial Composition of the Small Intestine

Upon sacrifice, the contents of the small intestine from 4 mice in the control group and 3 mice from the ALA-rich diet group were collected. Genomic DNA was purified from these digesta samples with the NucleoSpin Soil kit (Macherey-Nagel GmbH & Co.KG, Düren, Germany). Targeted sequencing of the 16S rRNA marker gene was employed in order to investigate the microbiome composition of the small intestine under the different dietary conditions. The V4-V5 hypervariable region of the bacterial 16S gene was amplified using specific PCR primers 515F-Y and 909R [28,29,30]. Paired-end sequencing was performed on the MiSeq Illumina platform by StarSEQ GmbH (Mainz, Germany). Subsequently, 16S data were processed with mothur v1.40.5 [31] following the MiSeq standard operating procedure [32]. Paired end-reads were merged into contigs, sequences with any ambiguous bases were removed and maximum homopolymer length was set to 8. Chimeric sequences were identified and removed with the VSEARCH algorithm within mothur [33]. Sequences were aligned to the SILVA database [34] and clustered to operational taxonomic units (OTUs) at 97% similarity. Taxonomic assignment was facilitated with the Ribosomal Database Project v9 [35] and OTUs with non-bacterial or unknown taxonomy were removed. Data were further analyzed and graphically represented with the help of the phyloseq R package v1.26.1 [36]. α-diversity was evaluated using the observed richness and Shannon index. For this analysis, each sample was normalized to the smallest library size by drawing a random subset of sequences with replacement. This step was repeated 100 times and the average values over all runs were reported as the final estimates for α-diversity. β-diversity was analyzed by computing the Bray-Curtis dissimilarity and the Jaccard binary distance. Canonical analysis of principal coordinates (CAP) [37], a constrained ordination procedure belonging to the same class of ordination methods as the unconstrained principal component analysis (PCA) [38] was employed to visualize results and investigate the multivariate hypothesis if an ALA diet significantly influences the microbial composition of the small intestine. Univariate differential abundance analysis was performed using the DESeq2 R package v1.22.2 [39]. For this purpose, OTUs were binned into phylotypes on the genus level. Taxa were considered to be differentially abundant if the adjusted p-value of the corresponding log2 fold change (FC) was *p* < 0.1. This more liberal threshold was selected because of the small sample size in an effort to increase power. P-values were adjusted for multiple comparisons with the Benjamini–Hochberg method.

### 2.5. Metagenome Prediction and Characterization

In order to gain insight into the potential functional profile of the small intestinal microbiome of mice under the different dietary conditions, we analyzed the 16S data using PICRUSt [40]. First, OTUs were re-assigned to a taxonomy using the Greengenes data base v13.5 [41]. Metagenome prediction was then performed up to Kyoto Encyclopedia of Genes and Genomes (KEGG) Orthology (https://www.genome.jp/kegg/ko.html) tier 3 using the PICRUSt online tool (http://huttenhower.sph.harvard.edu/galaxy/). Finally, results were visualized in STAMP v2.1.3 [42].

### 2.6. Statistical Analysis of Fatty Acid Profile and Small Intestinal Morphology

Data were graphically represented as mean values + standard error of the mean (SEM) and as individual values. Differences between two groups were statistically evaluated using unpaired t-test in case of normally distributed data or Mann–Whitney test when assumptions of normality were violated (according to the Kolmogorov Smirnov test). More than two groups were statistically compared with an ANOVA followed by Dunnett’s post-hoc test. P values were two-tailed and differences were considered statistically significant in case of *p* < 0.05. Statistical analysis was performed with GraphPad Prism version 6.07 (GraphPad Software Inc, San Diego, CA).

## 3. Results

### 3.1. Fatty Acid Profile of Different Diets

Perilla oil was chosen due to its very high content of ALA varying between 51%–64% [43,44,45,46]. In order to obtain an ALA-rich diet containing approximately 10% ALA, we mixed the control Altromin diet with perilla oil at a ratio of 8:2. The nutrient and energy content of the control diet were directly obtained from the feed producer (Table 1). The nutrient content of the ALA-rich diet was calculated based on the Altromin control chow by taking into account the addition of 20% fat. Furthermore, we experimentally confirmed the amount of ALA in the diet using GC-MS/MS. The fatty acid profile of the control and ALA-rich diet is shown in Table 1. ALA represented approx. 9.27% of the total ALA-rich diet and only 0.03% of the control chow. The addition of fat to the ALA-rich diet resulted in a calorie increase of approximately 30% relative to the control diet. In order to test the specificity of the effect of ALA-rich diet on gut morphology, we included a pro-inflammatory Western-type high-fat diet (HFD) (TD.88137, Envigo, Venray, Netherlands) with a comparable energy content. The composition and fatty acid profile of this HFD as obtained from the feed producer are summarized in Table 2.

### 3.2. ALA-Rich Diet Alters the Composition of the Microbiota in the Mid Small Intestine of Adult Mice

**Figure 2:**
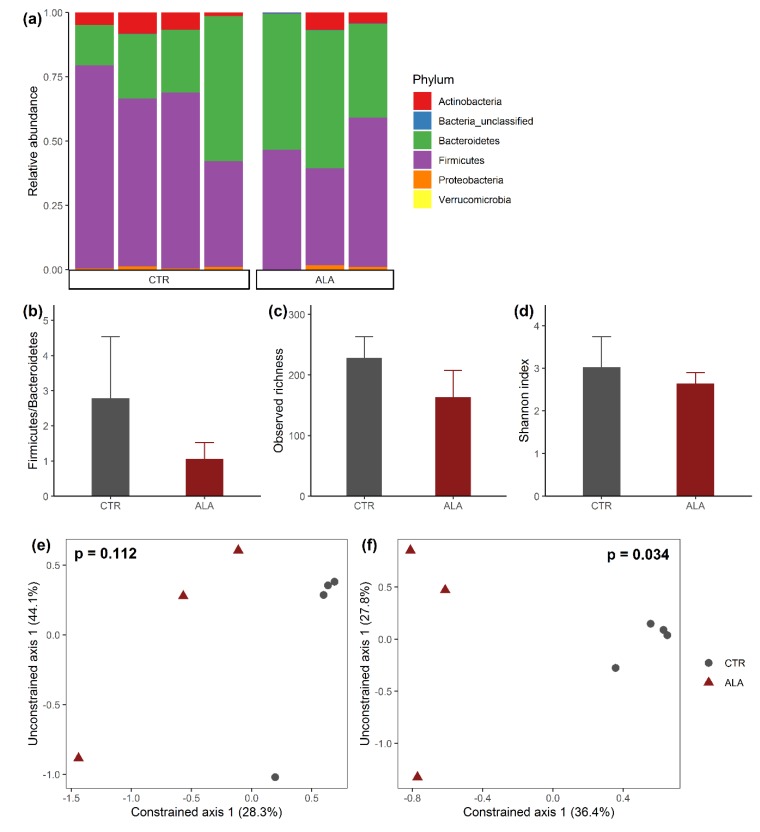
Microbial composition of the small intestine. (**a**) Bar plots show the relative abundance of bacterial phyla of individual animals in the control chow (CTR) or α-linolenic acid-rich diet group (ALA). (**b**) Firmicutes/Bacteroidetes ratio of CTR compared to ALA animals. α-diversity was investigated by estimating (**c**) the observed richness or (**d**) the Shannon index. Bar plots show mean + standard deviation for each measure per group. β-diversity was visualized using Canonical analysis of principle coordinates (CAP) based on (**e**) the Bray–Curtis dissimilarity or (**f**) binary Jaccard distance. P-values for the constrained axis from CAP were obtained using permutational analysis of variance (PERMANOVA) with 999 permutations. Since the treatment variable has only two levels (CTR or ALA), CAP produced only one constrained multivariate dimension. The percentage of the total inertia captured by each multivariate dimension is shown in brackets on the plots.

Microbial characterization of the commensal microbiota in the mid small intestine (jejunum) of mice resulted in identifying as many as 6747 unique OTUs. The majority of these OTUs were associated with the most dominant phyla Firmicutes and Bacteroidetes (Figure 2a). The average Firmicutes/Bacteroidetes ratio was considerably lower in animals fed with ALA-rich diet compared to the control diet (CTR) group (Figure 2b). The impact of ALA-rich diet feeding on microbiome diversity was in contrast to 16 weeks Western-type HFD feeding, which yielded an elevated Firmicutes/Bacteroides ratio [47]. Nevertheless, the difference was not statistically significant (unpaired *t*-test, *p* = 0.143) due to high intragroup variability. The remaining bacterial phyla represented in the ALA and CTR groups included Actinobacteria, Proteobacteria and Verrucomicrobia. The relative abundance of these phyla was comparably low under both feeding conditions. Microbiota composition of individual samples at the genus level is shown in Supplementary Figure S1.

ALA-rich diet was not associated with statistically significant changes in the estimates of α-diversity (Figure 2c,d). However, it is worth mentioning that the average observed richness was lower in the ALA group compared to CTR animals (mean difference = −141.14, 95% confidence interval (CI) = −141.5 to 10.7, *p* = 0.078). Analysis of β-diversity revealed that ALA mice were separated from the CTR group along the constrained multivariate dimension following CAP analysis based on the Bray–Curtis dissimilarity (Figure 2e) as well as the Jaccard distance (Figure 2f). Permutational analysis of variance (PERMANOVA) on the constrained axis confirmed that the dietary effect of ALA on microbial composition of the jejunum was statistically significant based on the Jaccard distance (*p* = 0.034). 

**Figure 3:**
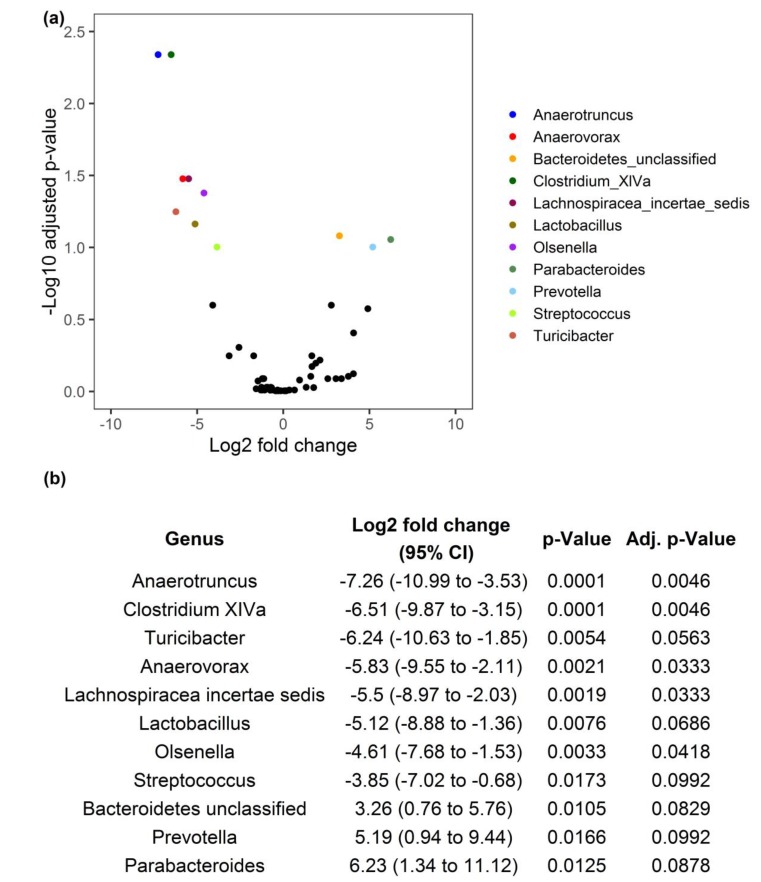
Univariate analysis of differentially abundant genera in the mid small intestine. (**a**) The plot shows the estimated log2 fold change (FC) of operational taxonomic unit (OTU) abundance in animals receiving an α-linolenic acid rich diet (ALA) relative to animals in the control chow group (CTR). The adjusted p-value for each FC is given as the negative decadic logarithm. More significant results appear as higher values on the y axis. Black dots indicate OTUs with non-significant FC whereas differentially abundant OTUs appear as colored dots. Negative FCs correspond to OTUs with a significantly reduced abundance in the ALA group compared to CTR animals. Positive FCs indicate OTUs with significantly increased abundance following ALA diet. Specific values for the log2 FC together with the corresponding 95% confidence interval (CI) and the raw and adjusted *p*-value are shown in (**b**).

After observing a shift in the multivariate profile of the commensal microbiota between both feeding conditions, we wanted to identify OTUs responsible for group differences at the genus level. For this purpose, we estimated the log2 fold changes (FC) of bacterial abundance in the small intestine of animals fed with an ALA-rich diet compared to control chow. This analysis was based on OTUs binned into phylotypes on the genus level, which resulted in 89 bins. Out of these, 11 phylotypes were differentially abundant (Figure 3). Interestingly, most of these OTUs were associated with a significantly lower abundance in the ALA group. All but one of the OTUs with significantly reduced abundance following ALA-rich diet were related to the Firmicutes phylum. The effect was most pronounced for OTUs associated with the genus *Anaerotruncus* (log2 FC = −7.26, 95% CI = −10.99 to −3.53, adj. *p* = 0.0046) and *Clostridium* cluster XIVa (log2 FC = −6.51, 95% CI = -9.57 to −3.15, adj. *p* = 0.0046). Both of these belong to the Clostridiales order and two other representatives of this order, namely *Anaerovorax* and Lachnospiraceae demonstrated significantly lower abundance in the ALA group as well. Two representatives of the lactic acid bacteria, namely *Lactobacillus* and *Streptococcus*, also decreased in abundance. An OTU related to the genus *Olsenella* (phylum Actinobacteria) was the only taxon, which did not belong to the Firmicutes phylum and had significantly reduced abundance in animals fed with an ALA-rich diet. In contrast, only three OTUs were enriched in the ALA group compared to CTR animals. All of these were related to the Bacteroidetes phylum. One OTU could not be classified beyond the phylum level. The remaining enriched OTUs were associated with the genera *Parabacteroides* (log2 FC = 6.23, 95% CI = 1.34 to 11.12, adj. *p* = 0.0125) and *Prevotella* (log2 FC = 5.19, 95% CI = 0.94 to 9.44, *p* = 0.0992).

### 3.3. ALA-Rich Diet Might Impact the Metagenome Profile of Commensal Microbiota in the Small Intestine

**Figure 4:**
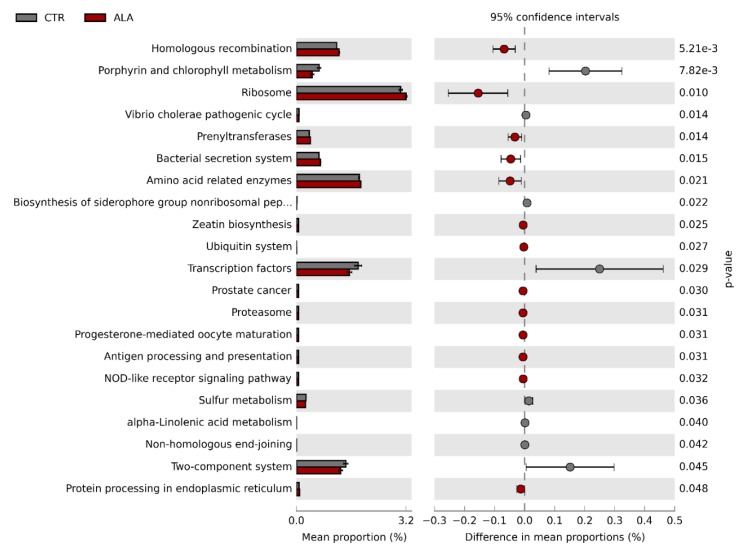
PICRUSt prediction of Kyoto Encyclopedia of Genes and Genomes (KEGG) orthology pathways predicted to be significantly different between the control group and animals receiving ALA-rich diet. Bar plots show the average proportion of sequences, which were predicted to be associated with the respective pathway. Circles correspond to the difference in mean proportions together with the 95% confidence interval. Red circles show pathways, which were enriched in the ALA group. Groups were compared statistically with an unpaired *t*-test. ALA: α-linolenic acid; CTR: control.

We employed the PICRUSt algorithm in order to predict the metagenome profile of the microbiome in the small intestine beyond its taxonomic composition. This analysis identified 21 KEGG orthology (KO) pathways, which might significantly differ between CTR and ALA dietary conditions (Figure 4). Out of these, 13 pathways were enriched following the ALA diet, though effect sizes (differences in mean proportions) were universally small. The most significantly enriched pathways in the ALA group included homologous recombination, ribosome, prenyltransferases, amino acid related enzymes and notably, the bacterial secretion system pathway. In contrast, eight pathways were predicted to be significantly downregulated in the ALA group. It is worth mentioning that the number of gene sequences predicted to be associated with the ALA metabolism pathway was significantly lower in the ALA group compared to controls (Figure 4).

### 3.4. ALA-Rich Diet Induces Changes in the Fatty Acid Composition of the Jejunum

**Figure 5:**
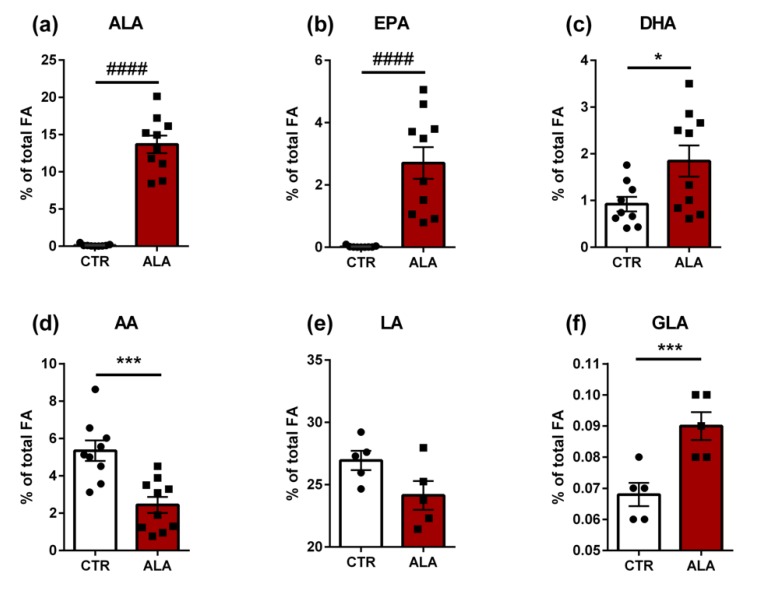
Impact of the ALA-rich diet on fatty acid composition of the mouse small intestine. Bar plots show mean values + standard error of the mean (SEM). *n* = 9 in control group, *n* =10 in ALA group for (**a**) ALA, (**b**) EPA, (**c**) DHA and (**d**) AA. *n* = 5 per group for (**e**) LA and (**f**) GLA. #### *p* < 0.0001, Mann-Whitney test; *** *p* < 0.001, * *p* < 0.05, unpaired *t*-test. ALA: α-linolenic acid; AA: arachidonic acid; CTR: control diet; DHA: docosahexanoic acid; EPA: eicosapentanoic acid; GLA: γ-linolenic acid; LA: linoleic acid.

Next, we investigated if increased dietary amounts of ALA are associated with changes in the fatty acid profile of the small intestine (Figure 5). The basal level of ALA in intestinal tissue as percentage of total fatty acids was around 0.1% in animals in the CTR group (Figure 5a). The ALA-rich diet led to a significant increase in the intestinal content of this fatty acid to approximately 13.6% (median difference = 13.91%, *p* < 0.0001). Similarly, levels of eicosapentanoic acid (EPA) were very low in the CTR group and significantly increased to approximately 2.7% following the ALA enriched-diet (*p* < 0.0001, Figure 5b). In contrast, the basal amount of docosahexanoic acid (DHA) was close to 0.9% (Figure 5c). The ALA diet was associated with a two-fold increase in DHA amount (mean difference = 0.92%, *p* = 0.027). The amount of arachidonic acid (AA) was significantly decreased in the ALA group compared to CTR animals (mean difference = −2.9%, 95% CI = −4.36% to −1.45%. *p* = 0.0006, Figure 5d). We did not observe a significant change in the amount of the ω-6 PUFA linoleic acid (Figure 5e). Furthermore, the ALA-rich diet led to a significant increase in the relative amount of γ-linolenic acid (Figure 5f).

We performed a more extensive profiling of fatty acid composition of the small intestinal tissue in a subset of animals (*n* = 5/group). ALA-rich diet significantly impacted the amount of 5 saturated fatty acids, lauric acid, myristic acid, pentadecanoic acid, palmitic acid and arachidic acid (Supplementary Table S1). The relative amount of all these saturated fatty acids was significantly lower in the ALA group compared to the CTR diet group. The levels of the monounsaturated fatty acids myristoleic acid, palmitoleic acid, oleic acid and 11-eicosanoic acid were also significantly reduced following ALA-rich diet. Furthermore, the relative amount of the ω-9 PUFA mead acid and the ω-6 PUFAs adrenic acid and osbond acid was significantly lower in the ALA group. In contrast, ALA-rich diet was associated with a significant increase in the levels of the ω-3 PUFAs stearidonic acid, eicosatrienoic acid, eicosatetranoic acid, docosatrienoic acid and docosapentanoic acid (Supplementary Table S1).

### 3.5. ALA-Rich Diet Shapes Small Intestinal Morphology

**Figure 6:**
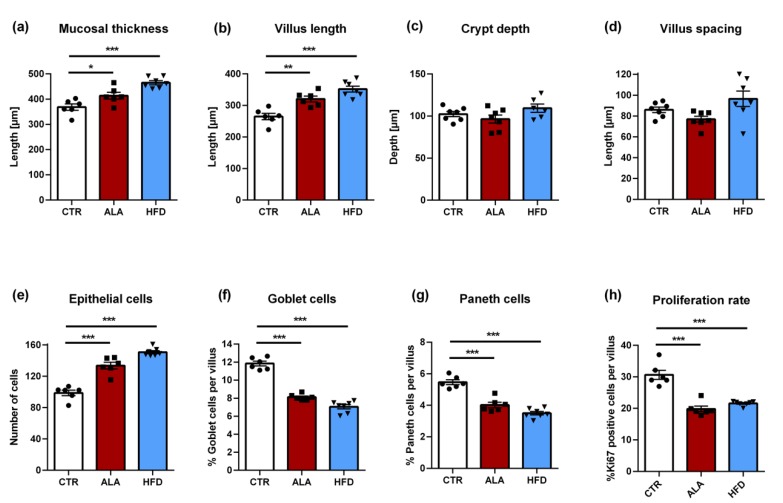
Impact of ALA-rich diet and Western-type high-fat diet (HFD) on villus morphology of the mid small intestine. Morphometric analysis of (**a**) mucosal thickness, (**b**) villus length, (**c**) crypt depth, and (**d**) villus spacing. Counts of (**e**) epithelial cells, (**f**) goblet cells, (**g**) Paneth cells. Based on Ki67 stained cells the proliferation rate was calculated (**h**). Bar plots show mean values + standard error of the mean (SEM). *n* = 6 in the CTR group, *n* = 6–7 in the ALA group and *n* = 7 in the HFD group. *** *p* < 0.001, ** *p* < 0.01, * *p* < 0.05, ANOVA followed by Dunnett’s post-hoc test. ALA: α-linolenic acid; CTR: control diet; HFD: Western-type high-fat diet.

**Figure 7:**
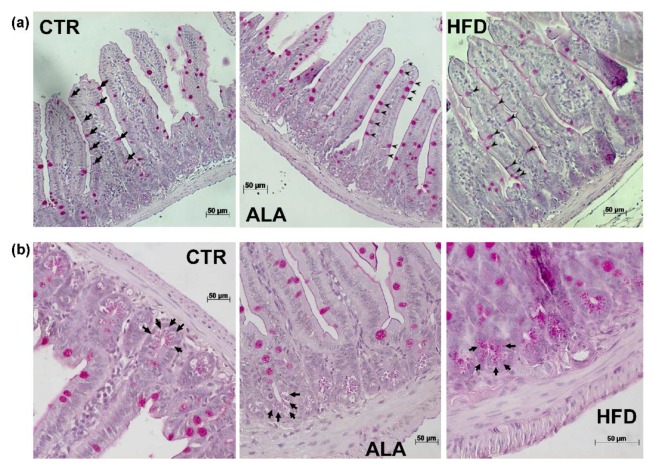
Representative images of Periodic acid-Schiff (PAS) staining of goblet and Paneth cells under different dietary conditions. Panel (**a**) shows PAS-stained goblet cells indicated by black arrows/arrow heads. PAS-stained Paneth cells in intestinal crypts are indicated with black arrows in panel (**b**). ALA: α-linolenic acid-rich diet; CTR: control diet; HFD: Western-type high-fat diet.

Since the PUFA-rich diet was suggested to affect the differentiation of the epithelial lineage [25], we investigated the impact of the ALA-rich diet on the morphology of the small intestine. In order to test the specificity of the ALA-rich diet effects, we also included a group of seven animals fed with a Western-type cholesterol-rich HFD. Both the ALA-rich and HFD were associated with significant increases in mucosal thickness and villus length compared to the CTR group and the effect was more pronounced in the HFD group (Figure 6a,b). Neither crypt depth nor villus spacing were significantly affected by the dietary intervention (Figure 6c,d). However, the ALA group showed a trend towards reduced villus spacing relative to the CTR group. The increased villus length was also reflected by the significantly higher number of epithelial cells in both the ALA and HFD groups relative to CTR animals (Figure 6e). However, the proportion of goblet and Paneth cells per villus was significantly reduced in the ALA-rich and HFD diet groups compared to control chow (Figure 6f,g). Representative images from PAS staining of goblet cells and Paneth cells are shown in Figure 7a,b, respectively. Finally, both the ALA-rich and Western-type HFD were accompanied by a significantly reduced proliferation rate, measured by the percentage of Ki67-positive cells per villus (Figure 6h).

## 4. Discussion

Our study demonstrates that ALA-rich high-fat diet leads to an altered composition of the commensal microbiota in the mid small intestine in healthy adult mice. This shift was more qualitative in nature as indicated by the statistically significant effect of diet in the CAP analysis based on the binary Jaccard index. In contrast, ordination analysis, based on the Bray–Curtis dissimilarity, which accounts for differences in bacterial abundance between groups, was not associated with a significant effect of diet. The small intestine of the ALA diet-fed mice showed increased total ALA content along with changed villus morphology. Specifically, villus length was increased both in ALA-rich and HFD-fed mice, accompanied with an increased mucosal thickness and number of epithelial cells. The epithelial proliferation rate as well as the proportion of goblet and Paneth cells was significantly reduced in the ALA-rich diet and HFD groups. 

With regards to the gut microbiome composition, we observed an increase in Bacteroidetes and a corresponding reduction in the relative abundance of Firmicutes following ALA-rich diet. The difference in the Firmicutes/Bacteroidetes ratio between groups was not statistically significant, but this is most likely due to small the sample size. Additionally, differential abundance analysis at the genus level confirmed these trends as most of the OTUs with reduced abundance in the ALA group were associated with the Firmicutes phylum. Remarkably, all enriched OTUs belonged to the Bacteroidetes group. Previous studies reported the opposite effect of an increase in Firmicutes abundance and a corresponding decrease in Bacteroidetes attributed to the Western-type high-fat diet [48,49]. Such a shift of the phylum composition has been linked to obesity in both mice and humans [49,50,51,52]. Mujico and colleagues observed that increased abundance of Firmicutes as well as *Clostridium* cluster XIVa and *Lactobacillus* was positively correlated with body weight in mice with diet induced obesity, whereas Bacteroidetes abundance was negatively correlated [49]. In our study, both *Lactobacillus* and *Clostridium* cluster XIVa associated OTUs showed decreased abundance in the ALA-rich diet group. Our results could therefore imply that an ALA-rich diet might have the potential to shift the microbiota composition to the lean phenotype. In agreement with this assumption, the Firmicutes/Bacteroidetes ratio was significantly decreased in type II diabetic patients following 6 months of EPA and DHA-rich diet compared to baseline [53]. Additionally, *Bacteroides-Prevotella* abundance was increased, which is similar to our finding of enhanced *Prevotella* growth in the ALA group. This suggests that ω-3 PUFA might exert similar effects on the gut microbiome. 

It is important to mention that previous studies investigated microbial composition of the colon or cecum whereas we focused on the mid small intestine, which might also contribute to differences in results. In this line of thought, we did not observe a change in the abundance of the *Bifidobacterium* genus, although *Bifidobacterium* strains were previously shown to produce ALA-derived metabolites [19]. This outcome might simply be due to the fact that these predominantly obligate anaerobic bacteria [54] are not abundant in the small intestine due to remaining small amounts of oxygen. Supporting this claim, we detected *Bifidobacteria*-associated OTUs only in one animal of the ALA group at a relative abundance of 0.02%. However, the abundance of OTUs associated with the *Lactobacillus* genus was also reduced following ALA-rich diet. It is important to mention that the ALA metabolism to conjugated fatty acids and growth inhibition was reported to be strain specific [19], whereas our analysis lacks the biological resolution to differentiate between individual bacterial strains. Additionally, Druart et al. detected the highest amounts of PUFA derived metabolites in the cecum and colon compared to jejunum and ileum, even though the small intestine is where the majority of fatty acid absorption takes place [55]. 

In our current study, we did not investigate the effects of the Western-type HFD on intestinal commensals as the influence of the HFD was addressed in previous work [48,49,51]. Furthermore, we tested the same HFD diet in a previous study, where we described a reduced Bacteroidetes and an increased Firmicutes abundance compared to control diet [47]. This finding is in agreement with previous reports. In contrast, we observed the opposite trend after feeding mice an ALA-rich diet. An ALA-rich diet was associated with a reduced abundance of *Anaerotruncus* and *Streptococcus*, whereas in our previous study the growth of these genera was stimulated by the Western-type HFD feeding [47]. Since both the ALA-rich diet and Western-type HFD had comparable caloric and fat content, these results suggest that the fatty acid pattern itself is an important factor shaping the composition of the intestinal microbiota. In support of this, we included a summary of further studies, which reported similar effects on microbiota composition following ALA or PUFA-rich diets in Supplementary Table S2.

Our predictive functional metagenome analysis implied that the ALA-rich diet might significantly impact a number of pathways, although effect sizes were generally small. Contrary to our expectations, we found the ALA metabolism pathway to be downregulated in the ALA group compared to control animals. This is probably due to the reduced abundance of OTUs associated with *Lactobacillus* and Lachnospiraceae as specific strains of these bacteria are reportedly able to metabolize ALA [21,22,23]. Furthermore, increased dietary amounts of ALA might interfere with the adaptive capabilities of gut bacteria since we observed a downregulation of the two-component system in the ALA-rich diet group. In line, we predicted significantly more sequences associated with the bacterial secretion system pathway compared to control animals, which might be an adaptive response of the commensal microbiota. Unsaturated fatty acids were previously reported to activate components of the bacterial secretion system of *Staphylococcus aureus* as part of its stress response [56,57]. Interestingly, the prostate cancer pathway was enriched in the ALA-group and a few reports have linked ALA levels with an increased risk for prostate cancer [58,59,60]. Nevertheless, other studies did not identify such association, so results remain controversial [16]. However, our findings regarding the functional metagenome are only based on in silico predictions and would have to be validated by whole metagenome shotgun sequencing.

Apart from the pronounced changes in gut microbiota composition, ALA-enriched diet resulted in an expected increase in ALA levels in the small intestinal tissue of mice. In line, the relative amount of EPA and DHA, oxylipins linked to reduced synthesis of inflammatory cytokines [61], was elevated in these tissues. The higher increase of EPA levels compared to DHA supports the conventional assumption that ALA is a better precursor for EPA than DHA in vivo [62]. In contrast to a previous study on the absorption of essential fatty acids in the rat jejunum [63], we noted a significant reduction in small intestinal AA levels in the ALA diet-fed group. Of note, the ALA diet-dependent up-regulation of fatty acid-binding protein 1 (FABP1; L-FABP) was reported in the small intestine of the C57BL/KsJ-db/dg obesity mouse model [64], demonstrating a direct influence of this essential fatty acid on the regulation of host metabolism. Overall, the ALA-rich diet was linked to a very consistent effect on the fatty acid profile of the small intestine as different classes of fatty acids were all regulated in the same direction. Namely, all significantly altered saturated fatty acids (SFA), monounsaturated fatty acids (MUFA), ω-6 PUFA and ω-9 PUFA demonstrated reduced levels in the ALA group whereas all significantly altered ω-3 PUFA showed an increased concentration. This indicates that an ALA-rich diet drives the fatty acid composition of the intestinal tissue towards an anti-inflammatory phenotype. For instance, decreasing the dietary ω-6/ ω-3 PUFA ratio was associated with significantly lower circulating levels of the pro-inflammatory marker interleukin-6 as well as non-high-density lipoprotein (non-HDL) cholesterol in low-density lipoprotein (LDL) receptor-deficient mice, which in turn led to attenuated aortic lesion formation [65]

In addition to influences on host metabolism, trophic effects on the mid small intestine were reported for bolus doses of essential fatty acids (i.e., ALA) [66]. In our study, the enrichment of ALA-derived metabolites in the small intestinal tissue was accompanied by the morphometric adaptation of the small intestine, as shown by increased mucosal thickness, increased villus length and an elevated number of epithelial cells per crypt-villus axis. These ALA-rich diet-induced elongated villus structures were associated with a vastly decreased epithelial cell proliferation rate. Considering that 4 weeks of Western-type HFD feeding yielded a similar gut mucosal morphology, the observed adaptation in gut morphology likely is a general feature of fat-rich diets. Noteworthy, the ALA-rich diet group showed a tendency of reduced villus spacing relative to the CTR group. Remarkably, our findings on dietary ALA, evoking reduced epithelial cell proliferation in the small intestine, are in support of the identified proliferation inhibitory effect of conjugated isomers of ALA (C18:3 c9, t11, c15 conjugated ALA), generated by *Bifidobacterium breve* National Collection of Industrial Food and Marine Bacteria (NCIMB) 702258 on the SW480 colon cancer cell line. Thus, we here provide direct evidence for the functional role of an ALA-enriched diet in influencing the small intestinal architecture. The functional involvement of HFD-induced dysbiosis in pathways driving morphometric adaptation of the small intestinal architecture is subject to further investigation. This aspect is in particular interesting since elongated villus structures and a decreased epithelial proliferation rate is a prominent trait of the germ-free mouse model [67]. The impact of a fat-rich diet on the morphogenetic adaptation of the small intestine may consequently have broad implications for nutrient uptake.

The nutritional impact on ALA-rich diet may also influence the differentiation of the secretory lineage. Specifically, we observed a marked decrease in the percentage of goblet and Paneth cells in the ALA group. This strongly suggests that additional gut developmental pathways are likewise influenced by fat-rich diet. In line with this, Benoit and colleagues demonstrated that ALA led to a significant decrease in the secretion of mucin 2 (MUC2) in a HT29-MTX cell line in vitro. Furthermore, the expression of the human atonal homolog 1 transcription factor (ATOH1), which is involved in goblet cell differentiation [68], was downregulated by ALA [25]. Interestingly, in the same study, oral administration of rapeseed oil to rat pups, which is rich in ω-3 PUFAs, was associated with reduced goblet cell numbers. An alternative mechanism through which a high-fat diet might influence intestinal morphology is via modifications in the composition of the commensal microbiota. Further studies such as feeding experiments in germ-free and conventionally raised mice would be necessary to investigate these hypotheses. As recent studies showed that modern nano-sized food additives are able to interact with both gut microbiota and biomolecules, it will be interesting to study the combined effects of nanoparticulates and macronutrients, such as ALA, in future studies [69,70,71,72,73].

### Limitation of the Analysis

The main limitation of our study is the small sample size for the analysis of the commensal microbiota composition in the small intestine. Due to the resulting low statistical power, our analysis was theoretically only able to detect large effect sizes. Therefore, an ALA-rich diet might result in additional changes in microbiome composition, which remained undetected. This limitation is partially offset by the statistical technique we employed. DESeq was previously shown to have increased sensitivity for differential abundance analysis in small samples compared to other methods [74]. Hence, our results on the effects of an ALA-rich diet on microbiota composition in the mouse small intestine should be verified in a larger cohort.

## 5. Conclusions

Our study on adult C57BL/6J mice revealed that an ALA-rich diet influences the microbiota composition and the predicted metagenome of the small intestine. Interestingly, the two-component system was downregulated, and the bacterial secretion system was upregulated in the ALA group, suggesting that an ALA-rich diet might affect bacterial adaptive responses. Moreover, we demonstrated the adaptation of gut morphology to an ALA-enriched diet, a trait that was also observed by feeding a Western-type HFD. Strikingly, the percentage of goblet and Paneth cells per crypt-villus axis was dramatically reduced under ALA-rich diet and Western-type HFD feeding conditions.

## Figures and Tables

**Table 1:** Composition and fatty acid profile of the standard Altromin 1814 laboratory chow and ALA-rich diet.

**Ingredient**	**Relative amount in control chow (% of diet)**	**Relative amount in ALA-Rich diet (% of diet)**
Proteins	17.61%	~14.1%
Fat	5.1%	~24%
Fiber	4.05%	~3.24%
Disaccharides	11.1%	~8.88%
Polysaccharides	47.2%	~37.76%
**Fatty acid**	**Relative amount (% of diet)**	**Relative amount (% of diet)**
Palmitic acid C-16:0	0.36%	4.1%
Stearic acid C-18:0	0.35%	5.55%
Oleic acid C-18:1 cis	0.93%	1.61%
Linoleic acid C-18:2 cis	3.3%%	2.96%
**α-Linolenic acid C-18:3 n3**	0.03%	9.27%
γ-Linolenic acid C18:3 n6	0.0002%	0.05%
Arachidic acid C-20:0	0.04%	0.21%
Eicosanoic acid C-20:1	0.01%	0.04%
Behenic acid C-22:0	0.04%	0.04%
Erucic acid C-22:1	0.02%	0.14%
Lignoceric acid C-24:0	0.01%	0.02%
Metabolizable energy	3518 kcal/kg	~4582 kcal/kg

**Table 2:** Composition and fatty acid profile of the pro-inflammatory TD.88137 high-fat Western diet.

**Ingredient**	**Relative amount (% of diet)**
Proteins	17.3%
Carbohydrates	48.5%
Fat	21.2%
**Fatty acid**	**Relative amount (% of diet)**
Saturated fat	13.1%
C-16:1	0.323%
Oleic acid C-18:1 cis	4.43%
C-18:1 isomers	0.85%
Linoleic acid C-18:2 cis	0.49%
C-18:2 isomers	0.28%
**α-Linolenic acid C18:3 n3**	0.15%
Metabolizable energy	4500 kcal/kg

## Supplementary Materials

The following are available online at https://www.mdpi.com/2072-6643/12/3/732/s1, Table S1. Fatty acid profile of intestinal tissue of mice receiving α-linolenic acid-rich diet or control diet; Table S2. Studies reporting similar effects of ALA- or PUFA-rich diet on microbiome composition; Figure S1. Composition of the small intestine microbiota at the genus level.

## References

[1] Caligiuri S.P.B., Parikh M., Stamenkovic A., Pierce G.N., Aukema H.M. (2017). Dietary modulation of oxylipins in cardiovascular disease and aging. Am. J. Physiol. Heart Circ. Physiol..

[2] Carroll A.E., Hwang D.H. (1980). Decreased formation of porstaglandins derived from arachidonic acid by dietary linolenate in rats. Am. J. Clin. Nutr..

[3] Horii T., Satouchi K., Kobayashi Y., Saito K., Watanabe S., Yoshida Y., Okuyama H. (1991). Effect of dietary alpha-linolenate on platelet-activating factor production in rat peritoneal polymorphonuclear leukocytes. J. Immunol..

[4] Thies F., Miles E.A., Nebe-von-Caron G., Powell J.R., Hurst T.L., Newsholme E.A., Calder P.C. (2001). Influence of dietary supplementation with long-chain n−3 or n−6 polyunsaturated fatty acids on blood inflammatory cell populations and functions and on plasma soluble adhesion molecules in healthy adults. Lipids.

[5] Caligiuri S.P.B., Aukema H.M., Ravandi A., Guzman R., Dibrov E., Pierce G.N. (2014). Flaxseed consumption reduces blood pressure in patients with hypertension by altering circulating oxylipins via an α-linolenic acid-induced inhibition of soluble epoxide hydrolase. Hypertension.

[6] Caligiuri S.P.B., Rodriguez-Leyva D., Aukema H.M., Ravandi A., Weighell W., Guzman R., Pierce G.N. (2016). Dietary flaxseed reduces central aortic blood pressure without cardiac involvement but through changes in plasma oxylipins. Hypertension.

[7] Kulkarni P.S., Eakins K.E. (1977). The enzymatic conversion of prostaglandin endoperoxides to thromboxane-A2-like activity by human iris microsomes. Prostaglandins.

[8] Ibrahim A., Aziz M., Hassan A., Mbodji K., Collasse E., Coëffier M., Bounoure F., Savoye G., Déchelotte P., Marion-Letellier R. (2012). Dietary α-linolenic acid–rich formula reduces adhesion molecules in rats with experimental colitis. Nutrition.

[9] Reifen R., Karlinsky A., Stark A.H., Berkovich Z., Nyska A. (2015). α-Linolenic acid (ALA) is an anti-inflammatory agent in inflammatory bowel disease. J. Nutr. Biochem..

[10] Trebble T.M., Pearl D.S., Eiden M., Masoodi M., Brümmer B.J., Gullick D., Mills G., Brown J.F., Shute J.K., Mckeever T.M. (2014). Altered colonic mucosal availability of n-3 and n-6 polyunsaturated fatty acids in ulcerative colitis and the relationship to disease activity. J. Crohns Colitis.

[11] Tyagi A., Kumar U., Reddy S., Santosh V.S., Mohammed S.B., Ehtesham N.Z., Ibrahim A. (2012). Attenuation of colonic inflammation by partial replacement of dietary linoleic acid with α-linolenic acid in a rat model of inflammatory bowel disease. Br. J. Nutr..

[12] Narisawa T., Takahashi M., Kotanagi H., Kusaka H., Yamazaki Y., Koyama H., Fukaura Y., Nishizawa Y., Kotsugai M., Isoda Y. (1991). Inhibitory effect of dietary perilla oil rich in the n-3 polyunsaturated fatty acid alpha-linolenic acid on colon carcinogenesis in rats. Jpn. J. Cancer Res. Gann.

[13] Tian Y., Wang H., Yuan F., Li N., Huang Q., He L., Wang L., Liu Z. (2016). Perilla oil has similar protective effects of fish oil on high-fat diet-induced nonalcoholic fatty liver disease and gut dysbiosis. BioMed Res. Int..

[14] Patterson E., O’Doherty R.M., Murphy E.F., Wall R., O’Sullivan O., Nilaweera K., Fitzgerald G.F., Cotter P.D., Ross R.P., Stanton C. (2014). Impact of dietary fatty acids on metabolic activity and host intestinal microbiota composition in C57BL/6J mice. Br. J. Nutr..

[15] Salsinha A.S., Pimentel L.L., Fontes A.L., Gomes A.M., Rodríguez-Alcalá L.M. (2018). Microbial production of conjugated linoleic acid and conjugated linolenic acid relies on a multienzymatic system. Microbiol. Mol. Biol. Rev..

[16] Hennessy A.A., Ross R.P., Devery R., Stanton C. (2011). The health promoting properties of the conjugated isomers of α-linolenic acid. Lipids.

[17] Yuan G.-F., Chen X.-E., Li D. (2014). Conjugated linolenic acids and their bioactivities: A review. Food Funct..

[18] Fontes A.L., Pimentel L.L., Simões C.D., Gomes A.M.P., Rodríguez-Alcalá L.M. (2017). Evidences and perspectives in the utilization of CLNA isomers as bioactive compounds in foods. Crit. Rev. Food Sci. Nutr..

[19] Gorissen L., Raes K., Weckx S., Dannenberger D., Leroy F., De Vuyst L., De Smet S. (2010). Production of conjugated linoleic acid and conjugated linolenic acid isomers by Bifidobacterium species. Appl. Microbiol. Biotechnol..

[20] Hennessy A.A., Barrett E., Paul Ross R., Fitzgerald G.F., Devery R., Stanton C. (2012). The production of conjugated α-linolenic, γ-linolenic and stearidonic acids by strains of Bifidobacteria and Propionibacteria. Lipids.

[21] Kishino S., Ogawa J., Ando A., Shimizu S. (2003). Conjugated α-linolenic acid production from α-linolenic acid by Lactobacillus plantarum AKU 1009a. Eur. J. Lipid Sci. Technol..

[22] Ogawa J., Kishino S., Ando A., Sugimoto S., Mihara K., Shimizu S. (2005). Production of conjugated fatty acids by lactic acid bacteria. J. Biosci. Bioeng..

[23] Druart C., Bindels L.B., Schmaltz R., Neyrinck A.M., Cani P.D., Walter J., Ramer-Tait A.E., Delzenne N.M. (2015). Ability of the gut microbiota to produce PUFA-derived bacterial metabolites: Proof of concept in germ-free versus conventionalized mice. Mol. Nutr. Food Res..

[24] Ohue-Kitano R., Yasuoka Y., Goto T., Kitamura N., Park S.-B., Kishino S., Kimura I., Kasubuchi M., Takahashi H., Li Y. (2017). α-Linolenic acid–derived metabolites from gut lactic acid bacteria induce differentiation of anti-inflammatory M2 macrophages through G protein-coupled receptor 40. FASEB J..

[25] Benoit B., Kayal F., Bruno J., Estienne M., Plaisancié P., Debard C., Ducroc R. (2015). Saturated and unsaturated fatty acids differently modulate colonic goblet cells in vitro and in rat pups. J. Nutr..

[26] Konieczka P., Barszcz M., Choct M., Smulikowska S. (2018). The interactive effect of dietary n-6: n-3 fatty acid ratio and vitamin E level on tissue lipid peroxidation, DNA damage in intestinal epithelial cells, and gut morphology in chickens of different ages. Poult. Sci..

[27] Schober Y., Wahl H.G., Renz H., Nockher W.A. (2017). Determination of red blood cell fatty acid profiles: Rapid and high-confident analysis by chemical ionization-gas chromatography-tandem mass spectrometry. J. Chromatogr. B.

[28] Walters W., Hyde E.R., Berg-Lyons D., Ackermann G., Humphrey G., Parada A., Gilbert J.A., Jansson J.K., Caporaso J.G., Fuhrman J.A. (2015). Improved bacterial 16S rRNA gene (V4 and V4-5) and fungal internal transcribed spacer marker gene primers for microbial community surveys. MSystems.

[29] Parada A.E., Needham D.M., Fuhrman J.A. (2016). Every base matters: Assessing small subunit rRNA primers for marine microbiomes with mock communities, time series and global field samples. Environ. Microbiol..

[30] Apprill A., McNally S., Parsons R., Weber L. (2015). Minor revision to V4 region SSU rRNA 806R gene primer greatly increases detection of SAR11 bacterioplankton. Aquat. Microb. Ecol..

[31] Schloss P.D., Westcott S.L., Ryabin T., Hall J.R., Hartmann M., Hollister E.B., Lesniewski R.A., Oakley B.B., Parks D.H., Robinson C.J. (2009). Introducing mothur: Open-source, platform-independent, community-supported software for describing and comparing microbial communities. Appl. Environ. Microbiol..

[32] Kozich J.J., Westcott S.L., Baxter N.T., Highlander S.K., Schloss P.D. (2013). Development of a dual-index sequencing strategy and curation pipeline for analyzing amplicon sequence data on the MiSeq Illumina sequencing platform. Appl. Environ. Microbiol..

[33] Rognes T., Flouri T., Nichols B., Quince C., Mahé F. (2016). VSEARCH: A versatile open source tool for metagenomics. PeerJ.

[34] Fuchs B.M., Quast C., Pruesse E., Peplies J., Knittel K., Ludwig W., Glöckner F.O. (2007). SILVA: A comprehensive online resource for quality checked and aligned ribosomal RNA sequence data compatible with ARB. Nucleic Acids Res..

[35] Kulam-Syed-Mohideen A.S., Chai B., McGarrell D.M., Cardenas E., Garrity G.M., Fish J., Tiedje J.M., Wang Q., Farris R.J., Marsh T. (2008). The Ribosomal Database Project: Improved alignments and new tools for rRNA analysis. Nucleic Acids Res..

[36] McMurdie P.J., Holmes S. (2013). phyloseq: An R package for reproducible interactive analysis and graphics of microbiome census data. PLoS ONE.

[37] Anderson M.J., Willis T.J. (2003). Canonical analysis of principal coordinates: A useful method of constrained ordination for ecology. Ecology.

[38] Todorov H., Fournier D., Gerber S. (2018). Principal components analysis: Theory and application to gene expression data analysis. Genom. Comput. Biol..

[39] Love M.I., Huber W., Anders S. (2014). Moderated estimation of fold change and dispersion for RNA-seq data with DESeq2. Genome Biol..

[40] Langille M.G.I., Zaneveld J., Caporaso J.G., McDonald D., Knights D., Reyes J.A., Clemente J.C., Burkepile D.E., Vega Thurber R.L., Knight R. (2013). Predictive functional profiling of microbial communities using 16S rRNA marker gene sequences. Nat. Biotechnol..

[41] McDonald D., Price M.N., Goodrich J., Nawrocki E.P., DeSantis T.Z., Probst A., Andersen G.L., Knight R., Hugenholtz P. (2012). An improved Greengenes taxonomy with explicit ranks for ecological and evolutionary analyses of bacteria and archaea. ISME J..

[42] Parks D.H., Tyson G.W., Hugenholtz P., Beiko R.G. (2014). STAMP: Statistical analysis of taxonomic and functional profiles. Bioinformatics.

[43] Shin H.-S., Kim S.-W. (1994). Lipid composition of perilla seed. J. Am. Oil Chem. Soc..

[44] Ding Y., Mokgolodi N.C., Hu Y., Shi L., Ma C., Liu Y.-J. (2012). Characterization of fatty acid composition from five perilla seed oils in China and its relationship to annual growth temperature. J. Med. Plants Res..

[45] Peirett P. (2011). Fatty acid content and chemical composition of vegetative parts of Perilla (Perilla frutescens) after different growth lengths. J. Med. Plants Res..

[46] Gwari G., Lohani H., Haider S.Z., Bhandari U., Chauhan N., Rawat D.S. (2014). Fatty acid and nutrient composition of Perilla (Perilla frutescens L.) accessions collected from Uttarakhand. Int. J. Phytopharm..

[47] Kiouptsi K., Jäckel S., Pontarollo G., Grill A., Kuijpers M.J.E., Wilms E., Weber C., Sommer F., Nagy M., Neideck C. (2019). The Microbiota Promotes Arterial Thrombosis in Low-Density Lipoprotein Receptor-Deficient Mice. MBio.

[48] Hildebrandt M.A., Hoffmann C., Sherrill-Mix S.A., Keilbaugh S.A., Hamady M., Chen Y.-Y., Knight R., Ahima R.S., Bushman F., Wu G.D. (2009). High-fat diet determines the composition of the murine gut microbiome independently of obesity. Gastroenterology.

[49] Mujico J.R., Baccan G.C., Gheorghe A., Díaz L.E., Marcos A. (2013). Changes in gut microbiota due to supplemented fatty acids in diet-induced obese mice. Br. J. Nutr..

[50] Ley R.E., Bäckhed F., Turnbaugh P., Lozupone C.A., Knight R.D., Gordon J.I. (2005). Obesity alters gut microbial ecology. Proc. Natl. Acad. Sci. USA.

[51] Ley R.E., Turnbaugh P.J., Klein S., Gordon J.I. (2006). Human gut microbes associated with obesity. Nature.

[52] Turnbaugh P.J., Ley R.E., Mahowald M.A., Magrini V., Mardis E.R., Gordon J.I. (2006). An obesity-associated gut microbiome with increased capacity for energy harvest. Nature.

[53] Balfegó M., Canivell S., Hanzu F.A., Sala-Vila A., Martínez-Medina M., Murillo S., Mur T., Ruano E.G., Linares F., Porras N. (2016). Effects of sardine-enriched diet on metabolic control, inflammation and gut microbiota in drug-naïve patients with type 2 diabetes: A pilot randomized trial. Lipids Health Dis..

[54] Biavati B., Vescovo M., Torriani S., Bottazzi V. (2000). Bifidobacteria: History, ecology, physiology and applications. Ann. Microbiol..

[55] Druart C., Neyrinck A.M., Vlaeminck B., Fievez V., Cani P.D., Delzenne N.M. (2014). Role of the lower and upper intestine in the production and absorption of gut microbiota-derived PUFA metabolites. PLoS ONE.

[56] Lopez M.S., Tan I.S., Yan D., Kang J., McCreary M., Modrusan Z., Austin C.D., Xu M., Brown E.J. (2017). Host-derived fatty acids activate type VII secretion in Staphylococcus aureus. Proc. Natl. Acad. Sci. USA.

[57] Kenny J.G., Ward D., Josefsson E., Jonsson I.-M., Hinds J., Rees H.H., Lindsay J.A., Tarkowski A., Horsburgh M.J. (2009). The Staphylococcus aureus response to unsaturated long chain free fatty acids: Survival mechanisms and virulence implications. PLoS ONE.

[58] Katan M.B., Zock P.L., Brouwer I.A. (2004). Dietary α-linolenic acid is associated with reduced risk of fatal coronary heart disease, but increased prostate cancer risk: A meta-analysis. J. Nutr..

[59] Ramon J.M., Bou R., Romea S., Alkiza M.E., Jacas M., Ribes J., Oromi J. (2000). Dietary fat intake and prostate cancer risk: A case–control study in Spain. Cancer Causes Control.

[60] De Stéfani E., Deneo-Pellegrini H., Boffetta P., Ronco A., Mendilaharsu M. (2000). α-linolenic acid and risk of prostate cancer: A case-control study in Uruguay. Cancer Epidemiol. Prev. Biomark..

[61] Dessì M., Noce A., Bertucci P., Manca di Villahermosa S., Zenobi R., Castagnola V., Addessi E., Di Daniele N. (2013). Atherosclerosis, dyslipidemia, and inflammation: The significant role of polyunsaturated fatty acids. ISRN Inflamm..

[62] Burdge G.C. (2006). Metabolism of α-linolenic acid in humans. Prostaglandins Leukot. Essent. Fat. Acids.

[63] Punchard N.A., Green A.T., Mullins J.G., Thompson R.P.H. (2000). Analysis of the intestinal absorption of essential fatty acids in vivo in the rat. Prostaglandins Leukot. Essent. Fat. Acids.

[64] Nagasawa A., Suzuki J., Hase T., Murase T., Wakisaka T., Tokimitsu I. (2002). Dietary α-linolenic acid–rich diacylglycerols reduce body weight gain accompanying the stimulation of intestinal β-oxidation and related gene expressions in C57BL/KsJ-db/db mice. J. Nutr..

[65] Wang S., Wu D., Matthan N.R., Lamon-Fava S., Lecker J.L., Lichtenstein A.H. (2009). Reduction in dietary omega-6 polyunsaturated fatty acids: Eicosapentaenoic acid plus docosahexaenoic acid ratio minimizes atherosclerotic lesion formation and inflammatory response in the LDL receptor null mouse. Atherosclerosis.

[66] Jenkins A.P., Thompson R.P. (1993). Does the fatty acid profile of dietary fat influence its trophic effect on the small intestinal mucosa?. Gut.

[67] Abrams G.D., Baurer H., Sprinz H. (1963). Influence of the normal flora on mucosal morphology and cellular renewal in the ileum. A comparison of germ-free and conventional mice. Lab. Investig..

[68] Zheng X., Tsuchiya K., Okamoto R., Iwasaki M., Kano Y., Sakamoto N., Nakamura T., Watanabe M. (2011). Suppression of hath1 gene expression directly regulated by hes1 via notch signaling is associated with goblet cell depletion in ulcerative colitis. Inflamm. Bowel Dis..

[69] Docter D., Westmeier D., Markiewicz M., Stolte S., Knauer S.K., Stauber R.H. (2015). The nanoparticle biomolecule corona: Lessons learned*—*Challenge accepted. Chem. Soc. Rev..

[70] Westmeier D., Hahlbrock A., Reinhardt C., Frohlich-Nowoisky J., Wessler S., Vallet C., Poschl U., Knauer S.K., Stauber R.H. (2018). Nanomaterial-microbe cross-talk: Physicochemical principles and (patho)biological consequences. Chem. Soc. Rev..

[71] Siemer S., Hahlbrock A., Vallet C., McClements D.J., Balszuweit J., Voskuhl J., Docter D., Wessler S., Knauer S.K., Westmeier D. (2018). Nanosized food additives impact beneficial and pathogenic bacteria in the human gut: A simulated gastrointestinal study. NPJ Sci. Food.

[72] Stauber R.H., Siemer S., Becker S., Ding G.B., Strieth S., Knauer S.K. (2018). Small Meets Smaller: Effects of Nanomaterials on Microbial Biology, Pathology, and Ecology. ACS Nano.

[73] Siemer S., Westmeier D., Vallet C., Steinmann J., Buer J., Stauber R.H., Knauer S.K. (2018). Breaking resistance to nanoantibiotics by overriding corona-dependent inhibition using a pH-switch. Mater. Today.

[74] Weiss S., Xu Z.Z., Peddada S., Amir A., Bittinger K., Gonzalez A., Lozupone C., Zaneveld J.R., Vázquez-Baeza Y., Birmingham A. (2017). Normalization and microbial differential abundance strategies depend upon data characteristics. Microbiome.

