# Supplementary material for: α-Linolenic Acid-Rich Diet Influences Microbiota Composition and Villus Morphology of the Mouse Small Intestine
Source: Nutrients. 2020 Mar 11;12(3):732. doi: 10.3390/nu12030732 (PMC7146139; doi:10.3390/nu12030732)
Supplement: Supplementary file 1 [file nutrients-12-00732-s001.pdf]

α-Linolenic acid-rich diet influences microbiota composition and villus morphology of the mouse small intestine

Hristo Todorov, Bettina Kollar, Franziska Bayer, Inês Brandão, Amrit Mann, Julia Mohr, Giulia Pontarollo, Henning Formes, Roland Stauber, Jens M. Kittner, Kristina Endres, Bernhard Watzer, Wolfgang Andreas Nockher, Felix Sommer, Susanne Gerber, Christoph Reinhardt

Table S1. Fatty acid profile of intestinal tissue of mice receiving α-linolenic acid-rich diet or control diet.

Fatty acid	Control diet (mean ± SEM)	ALA diet (mean ± SEM)	p-value
C8:0 Caprylic acid	0.00 ± 0.00	0.00 ± 0.00	n.s.
C10:0 Capric acid	0.00 ± 0.00	0.00 ± 0.00	n.s.
C11:0 Undecylic acid	0.00 ± 0.00	0.00 ± 0.00	n.s.
C12:0 Lauric acid	0.026 ± 0.007	0.006 ± 0.002	0.032#
C13:0 Tridecylic acid	0.00 ± 0.00	0.00 ± 0.00	n.s.
C14:0 Myristic acid	1.84 ± 0.24	0.45 ± 0.06	0.003**
C14:1 Myristoleic acid	0.038 ± 0.010	0.002 ± 0.002	0.008##
C15:0 Pentadecanoic acid	0.086 ± 0.002	0.07 ± 0.003	0.024#
C15:1 Pentadecenoic acid	0.00 ± 0.00	0.00 ± 0.00	n.s.
C16:0 Palmitic acid	31.43 ± 1.01	14.82 ± 0.54	<0.0001***
C16:1 Palmitoleic acid	4.50 ± 0.96	0.41 ± 0.09	0.013*
C17:0 Margaric acid	0.17 ± 0.02	0.20 ± 0.02	n.s.
C18:0 Stearic acid	9.51 ± 1.05	12.54 ± 1.10	n.s.
C18:1 trans Elaidic acid	0.048 ± 0.024	0.042 ± 0.004	n.s.
C18:1 cis Oleic acid	15.16 ± 0.68	7.20 ± 0.88	<0.0001***
C18:2 trans Linolelaidic acid	0.016 ± 0.016	0.002 ± 0.002	n.s.
C20:0 Arachidic acid	0.32 ± 0.03	0.25 ± 0.01	0.048*
C20:1 11-Eicosanoic acid	0.44 ± 0.03	0.24 ± 0.02	0.016#
C21:0 Heneicosylic acid	0.01 ± 0.0	0.008 ± 0.002	n.s.
C18:4 n3 Stearidonic acid	0.37 ± 0.14	10.64 ± 0.63	0.008##
C20:2 Eicosadienoic acid	0.12 ± 0.011	0.13 ± 0.013	n.s.

C22:0 Behenic acid	0.08 ± 0.014	0.056 ± 0.006	n.s.
C20:3 n9 Mead acid	1.13 ± 0.07	0.61 ± 0.09	0.002**
C20:3 n3 Eicosatrienoic acid	0.00 ± 0.00	0.16 ± 0.02	<0.0001***
C23:0 Tricosylic acid	1.34 ± 0.38	1.15 ± 0.55	n.s.
C22:2 Docosadienoic acid	0.00 ± 0.00	0.002 ± 0.002	n.s.
C24:0 Lignoceric acid	0.048 ± 0.007	0.046 ± 0.006	n.s.
C20:4 n3 Eicosatetranoic acid	0.042 ± 0.042	2.64± 0.53	0.008##
C22:3 Docosatrienoic acid	0.00 ± 0.00	5.49 ± 0.36	0.008##
C24:1 Nervonic acid	0.036 ± 0.005	0.04 ± 0.004	n.s.
C22:4 n6 Adrenic acid	0.64 ± 0.08	0.094 ± 0.011	0.002**
C22:5 n6 Osbond acid	0.45 ± 0.05	0.012 ± 0.002	0.008##
C22:5 n3 Docosapentanoic acid	0.056 ± 0.019	1.29 ± 0.19	0.008##

n.s. not significant *** p<0.001, ** p<0.01, *p<0.05 unpaired t-test; ### p<0.001, ## p<0.01, #p<0.05 Mann-Whitney U test.

Table S2. Studies reporting similar effects of ALA- or PUFA-rich diet on microbiome composition

Study	Study population	Diet	ALA/PUFA effects on microbiome comparable to our study
Tial et al. 2016[1]	Male Sprague-Dawley rats, 8-9 weeks old at study start	1. Normal chow diet with 10kcal% fat for 16 weeks<br>2. Western style lard-rich diet with 45 kcal% fat<br>3. Fish oil rich diet with 10% fish oil<br>4. Perilla-oil rich diet with 5.5 % perilla oil	Reduced Firmicutes/Bacteroidetes ratio; enhanced *Prevotella* and *Parabacteroides* growth; reduced *Lactobacillus* growth
Wang et al. 2018[2]	8-week old spontaneously diabetic male KKay mice (DM) or age-matched C57L/6 mice (NC)	1. NC + normal chow<br>2. DM + control chow<br>3. DM + low dose perilla oil (0.67 g/kg/bw/d)<br>4. DM + middle dose perilla oil (1.33 g/kg/bw/d)<br>5. DM + high dose perilla oil (2 g/kg/bw/d)	Enhanced *Parabacteroides* growth; reduced abundance of Lachnospiraceae in low dose perilla oil group compared to DM control.
Power et al 2016[3]	4-week old male C57Bl/6 mice	1. Basal diet for 3 weeks<br>2. Basal diet supplemented with 10% whole ground flaxseed oil	Enhanced abundance of *Prevotella*
Patterson et al. 2014[4]	8-week old male C57Bl/6 mice	1. Low fat- high maize starch diet for 16 weeks<br>2. Low fat-high sucrose diet<br>3. High fat-palm oil	Reduced *Anaerotruncus* growth in flaxseed/fish oil group compared to low fat-high sucrose group

		4. High fat-olive oil<br>5. High fat-safflower oil<br>6. High fat-flaxseed/fish oil	
Pusceddu et al. 2015[5]	17-week old female Sprague-Dawley rats (maternally separated and non-separated animals)	1. Saline water<br>2 Low dose EPA/DHA (0.4 g/kg/day mixture with 80% EPA and 20%DHA)<br>3. High dose EPA/DHA (1 g/kg/day mixture with 80% EPA and 20%DHA)	Enhanced *Prevotella* growth in low dose and high dose EPA/DHA group compared to control group (non-separated animals)
Beilharz et al. 2016[6]	Male Srague-Dawley rats	1. Control diet (12 or 13 days)<br>2. Saturated fat diet (with lard as source)<br>3. PUFA rich diet (with sunflower oil as source)<br>4. Sugar diet	Reduced *Lactobacillus* growth in PUFA group compared to control diet

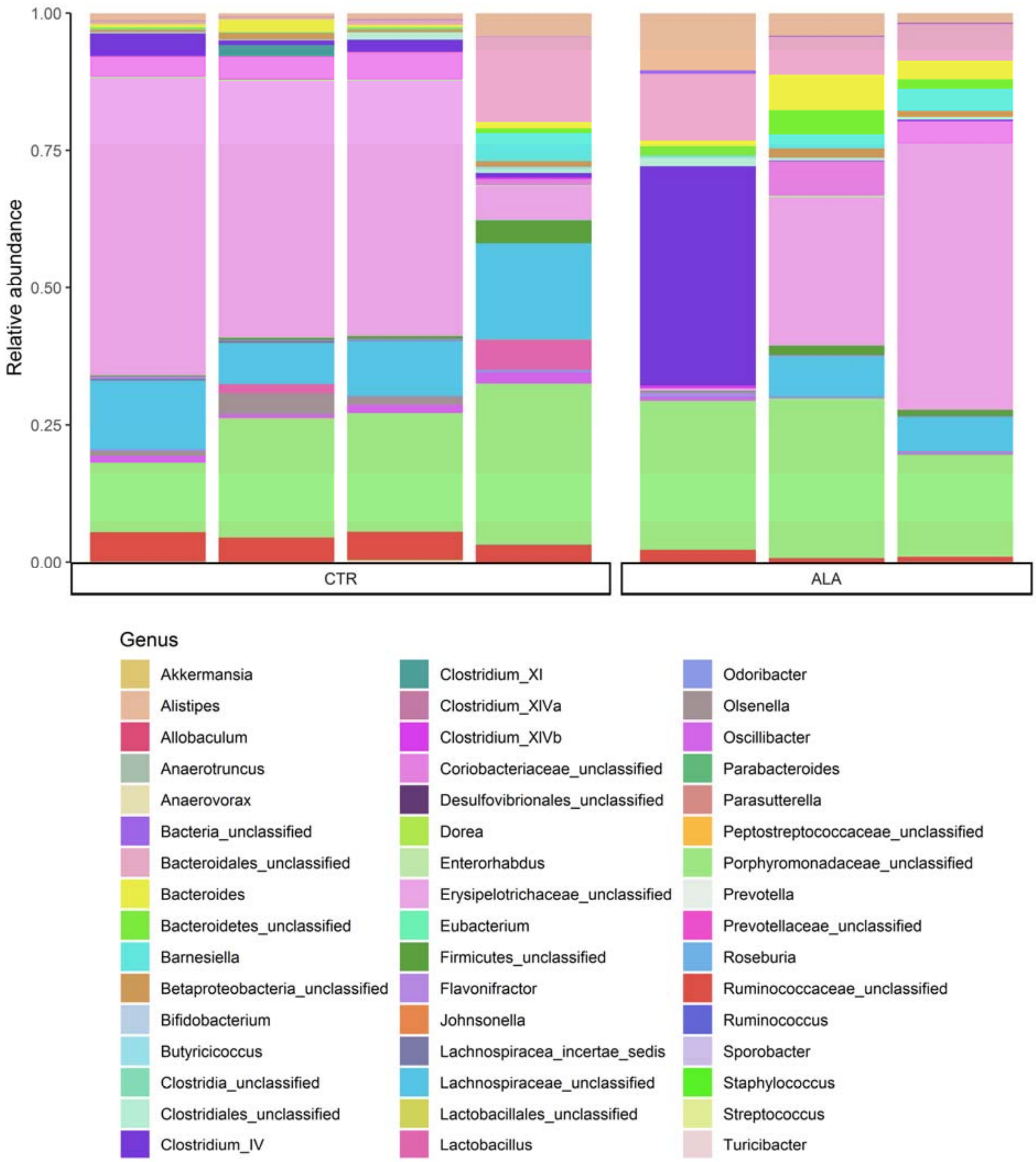

Figure S1. Composition of the small intestine microbiota at the genus level. Data are shown as relative abundance for each animal in the α-linolenic acid-rich diet group (ALA) or the control chow group (CTR).

References

1 Tian, Y. *et al.* Perilla oil has similar protective effects of fish oil on high-fat diet-induced nonalcoholic fatty liver disease and gut dysbiosis. *BioMed Research International* **2016**, 11, doi:10.1155/2016/9462571 (2016).

2 Wang, F. *et al.* Perilla Oil Supplementation Improves Hypertriglyceridemia and Gut Dysbiosis in Diabetic KKAy Mice. *Molecular nutrition & food research* **62**, e1800299-e1800299, doi:10.1002/mnfr.201800299 (2018).

3 Power, K. A. *et al.* Dietary flaxseed modulates the colonic microenvironment in healthy C57Bl/6 male mice which may alter susceptibility to gut-associated diseases. *The Journal of Nutritional Biochemistry* **28**, 61-69, doi:https://doi.org/10.1016/j.jnutbio.2015.09.028 (2016).

4 Patterson, E. *et al.* Impact of dietary fatty acids on metabolic activity and host intestinal microbiota composition in C57BL/6J mice. *British Journal of Nutrition* **111**, 1905-1917, doi:10.1017/S0007114514000117 (2014).

5 Pusceddu, M. M. *et al.* N-3 Polyunsaturated Fatty Acids (PUFAs) Reverse the Impact of Early-Life Stress on the Gut Microbiota. *PloS one* **10**, e0139721-e0139721, doi:10.1371/journal.pone.0139721 (2015).

6 Beilharz, J. E., Kaakoush, N. O., Maniam, J. & Morris, M. J. The effect of short-term exposure to energy-matched diets enriched in fat or sugar on memory, gut microbiota and markers of brain inflammation and plasticity. *Brain, Behavior, and Immunity* **57**, 304-313, doi:https://doi.org/10.1016/j.bbi.2016.07.151 (2016).
